# Comparison of Transcriptome Differences between Two Rice Cultivars Differing in Cadmium Translocation from Spike-Neck to Grain

**DOI:** 10.3390/ijms25073592

**Published:** 2024-03-22

**Authors:** Shouping Zhao, Qi Zhang, Wendan Xiao, De Chen, Jing Hu, Na Gao, Miaojie Huang, Xuezhu Ye

**Affiliations:** State Key Laboratory for Managing Biotic and Chemical Threats to the Quality and Safety of Agro-Products, Key Laboratory of Information Traceability for Agricultural Products, Institute of Agro-Product Safety and Nutrition, Zhejiang Academy of Agricultural Sciences, Hangzhou 310021, China; zhaosp@zaas.ac.cn (S.Z.); zhangq@zaas.ac.cn (Q.Z.); xiaowd@zaas.ac.cn (W.X.); chend@zaas.ac.cn (D.C.); hujing987@hotmail.com (J.H.); gaonagaona987@hotmail.com (N.G.); huangmj987@hotmail.com (M.H.)

**Keywords:** rice cultivars, RNA-seq, WGCNA, cadmium, transport efficiency, spike-neck

## Abstract

At present, the mechanism of varietal differences in cadmium (Cd) accumulation in rice is not well understood. Two rice cultivars, ZZY (high translocation-high grain Cd) and SJ18 (low translocation-low grain Cd), were used to analyze transcriptome differences in the spike-neck tissue in field trials. The results showed that, compared with ZZY, 22,367 differentially expressed genes (DEGs) were identified in SJ18, including 2941 upregulated and 19,426 downregulated genes. GO analysis enriched 59 downregulated terms, concerning 24 terms enriched for more than 1000 DEGs, including cellular and metabolic processes, biological regulation, localization, catalytic activity, transporter activity, signaling, etc. KEGG enrichment identified 21 significant downregulated pathways, regarding the ribosome, metabolic pathways, biosynthesis of secondary metabolism, signaling transduction, cell membrane and cytoskeleton synthesis, genetic information transfer, amino acid synthesis, etc. Weighted gene co-expression network analysis (WGCNA) revealed that these DEGs could be clustered into five modules. Among them, the yellow module was significantly related to SJ18 with hub genes related to OsHMA and OsActin, whereas the brown module was significantly related to ZZY with hub genes related to mitogen-activated protein kinase (MAPK), CBS, and glutaredoxin. This suggests that different mechanisms are involved in the process of spike-neck–grain Cd translocation among varieties. This study provides new insights into the mechanisms underlying differences in Cd transport among rice varieties.

## 1. Introduction

Land pollution is a global problem, particularly in developing countries. The high concentrations of Cd in paddy soils, its accumulation in rice, and its subsequent transport to humans through the food chain are global environmental problems [1]. Numerous studies have explored the mechanisms of Cd uptake, translocation, resistance, and tolerance in rice [2]. Simultaneously, several studies have been conducted on the regulation of Cd transfer from the soil to rice plants and its redistribution within them [3]. Among these, the use of low-Cd-accumulating genotypes to regulate Cd uptake in rice has been an environmentally friendly and practical tool, and the existence of differences in Cd accumulation between crop species and varieties within species, including rice, has been recognized [4,5]. However, these studies relied on the elucidation of the mechanisms involved in Cd variation among rice cultivars, including their related key traits and even functional genes.

The enrichment of rice Cd from roots to grains is a long and complex transport process disturbed by multiple factors, including crop variety, agronomic measures, soil conditions, and climatic conditions. In general, rice varieties with low Cd accumulation have distinctive characteristics in one or more respects, including agronomic traits, plant Cd uptake and translocation, and physiological and metabolic responses to Cd stress. Agronomic traits have been found to be related to the Cd accumulation capacity of crops, and the wheat grain Cd content is significantly and positively correlated with root dry biomass, but not with grain yield [6]. A significant positive correlation between rice yield and Cd concentration in grains has been noted [7], which means that screening low-cadmium-enriched, high-yielding rice varieties is not easy and is even more difficult in practical applications [4]. In addition, rice cultivars with low Cd accumulation also have low bioconcentration factors, which are mainly influenced by soil status and crop species [4,8]. Grain Cd is positively correlated with the total plant Cd accumulation and root-to-aboveground and aboveground-to-grain Cd distribution [9]. Cd retention in roots and subsequent low roots to stems, as well as from stems to brown rice, play an essential role in low-Cd rice lines [10].

These processes are associated with a series of complex physiological processes. In recent years, comparative transcriptomic analyses have been used to analyze the typical metabolic characteristics of low-Cd-accumulating varieties and their associated genes. Using this technique, low-Cd rice has been shown to have distinct characteristics in terms of metabolites, the biosynthesis of secondary metabolites, signaling pathways, and other responses to Cd stress [11]. In addition, cell wall biosynthesis in low-Cd rice varieties is more responsive to Cd stress, resulting in more Cd sequestration in the cell wall, and with the upregulation of sulfur and glutathione metabolism, more glutathione and phytochelatins are synthesized, resulting in more Cd compartments being chambered in the vacuoles [12]. In contrast, processes such as the redox state, ion transport across membranes, transcription factor regulation, signal transduction, synthesis, and metabolism of macromolecules and sulfur-containing compounds in rice are all related to Cd stress, and previous studies have identified a series of new functional genes [13]. Weighted gene co-expression network analysis (WGCNA) is a method of clustering and analyzing all differentially expressed genes based on gene expression chaining patterns and obtaining hub genes from transcriptome data. It has been used to mine key genes for abiotic stress tolerance in plants. For example, key processes and functional genes for drought, salt, and cold stress tolerance in rice have been identified [14,15,16]. Moreover, important physiological processes and the key genes responsible for differences in root Cd uptake capacity among rice varieties have been identified by comprehensive transcriptome and WGCNA analyses [17].

Although there have been many studies on varietal differences in Cd tolerance and accumulation, rice Cd accumulation is a long-distance soil–root–stem–grain transport process that is affected by many factors; thus, the mechanism of Cd accumulation in rice grains is not yet clear. As research progresses, more key traits need to be explored and more advanced analytical tools need to be utilized to reveal the key biological mechanisms of all aspects of Cd long-distance transport in low-accumulating rice varieties. In this study, two rice varieties with significantly different grain Cd contents under field conditions were used to elucidate the unique traits of low-grain Cd accumulation varieties in the redistribution of Cd in aboveground plants, especially the associated molecular basis and key genes, by transcriptomic and WGCNA approaches, aiming to provide new insights into the mechanisms affecting Cd accumulation among rice varieties, as well as to provide new directions for the screening and breeding of low-accumulating varieties.

## 2. Results

### 2.1. Cadmium and Its Transfer in Rice Plant

**Figure 1:**
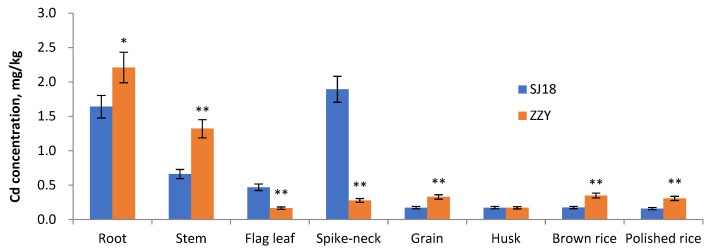
Cd concentrations in root, stem, flag leaf, spike-neck, grain, husk, brown rice, and polished rice of SJ18 and ZZY. Data are means ± SE of three replicates. Asterisks indicate significant differences from SJ18 at * *p* < 0.05 and ** *p* < 0.01 by Student’s *t*-test.

There were significant differences in Cd content between the two varieties in all plant parts, except for rice husks (Figure 1). The Cd in the roots, stems, grains, brown rice, and polished rice was significantly higher in ZZY than in SJ18, whereas the Cd in the flag leaf and spike-neck was significantly higher in SJ18 than in ZZY. This suggests that there are intraspecies differences in the process of Cd redistribution and translocation in the aboveground parts of the plant. In terms of root–stem Cd transfer efficiency, that of SJ18 (40.4%) was significantly lower than that of ZZY (59.7%), whereas no significant differences were detected between cultivars in terms of the stem–grain Cd transfer efficiencies, which were 26.1% and 24.8% in SJ18 and ZZY, respectively.

Accounting for the redistribution rates in aboveground plants, with the significantly lower Cd in flag leaf and spike-neck than SJ18, ZZY achieved a ratio of 1.97:1.18:1.00 for cadmium content in flag leaf, spike-neck, and rice grain; meanwhile, the corresponding ratio was 0.36:0.09:1.00 in SJ18. The significantly lower Cd in spike-neck of ZZY than in those of SJ18 indicated that ZZY achieved 118% spike-neck–grain Cd translocation efficiency, which was 9.13% in SJ18. This suggested that ZZY had a significantly stronger ability to transfer Cd from the spike-neck to the grain than SJ18, with a difference greater than ten times as much. Taken together, the high grain Cd of ZZY was related to its high root Cd and strong Cd translocation to the aboveground parts, while its strong Cd translocation from the spike-neck to the grain may contribute more to the higher grain Cd than that of SJ18.

The spike-neck is the connecting part of the rice spike to the stem, the upper part of which is the spike and the lower part is the stem, which plays a key role in Cd accumulation in rice grains. Considering the significant differences in Cd translocation from the spike-neck to the grain between the two varieties, a comparative analysis of the spike-neck gene expression profiles was carried out in this study. In addition, the distribution of Cd in the grains was noteworthy. Although the Cd content of the grain and polished or brown rice of both varieties was significantly higher in ZZY than in SJ18, no significant difference existed in husk Cd between varieties, suggesting that there were different mechanisms of Cd redistribution in the grains between varieties.

### 2.2. RNA-Seq Data, qRT-PCR Validation, and Function Annotation

**Figure 2:**
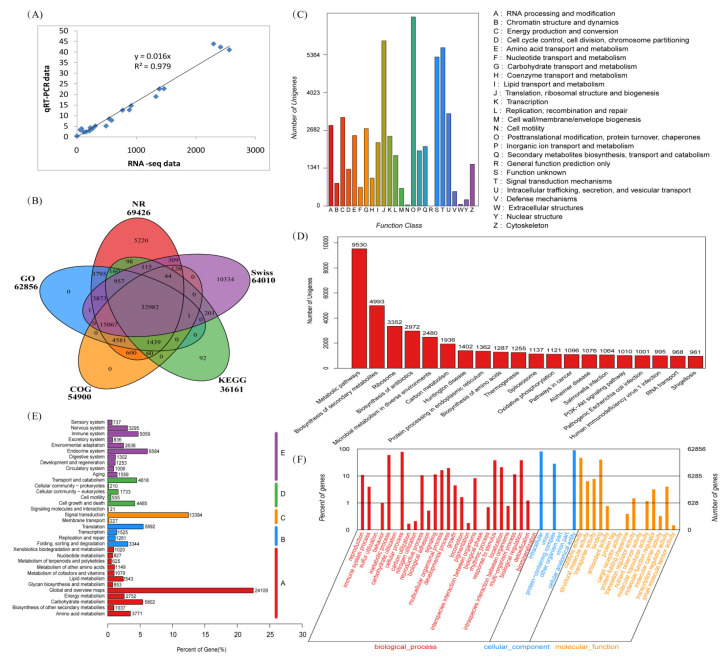
RNA-seq data validation by qRT-PCR and function annotation of assembled genes. (**A**) Linear correlation analysis of the expression level for the selected genes between RNA-seq and qRT-PCR results; (**B**) Venn diagram of all assembled genes versus NR, Swiss, KEGG, COG, and GO databases; (**C**) COG classification for all the assembled genes; (**D**) top 20 KEGG pathways annotated for all assembled genes; (**E**) category of all assembled genes functionally annotated in KEGG pathways; (**F**) GO functional annotation of all assembled genes.

From the six spike-neck samples, RNA-seq analysis obtained 34.8–45.7 million raw reads with a mean value of 38.9 million. After quality assessment, 22.3–36.4 million clean reads with a mean value of 30.8 million were used for subsequent de novo assembly (Supplemental Table S1). Of them, 15.6–32.0 million (mean value was 25.7 million) reads were successfully mapped to the reference transcriptome with a mapped rate of 70.0–90.0% (mean value of 82.5%). After filtration, the Q30 value was 89.7–90.7% (mean value of 90.1%) and the GC values ranged from 48.7% to 53.2% (mean value of 50.5%). These data suggest that the RNA-seq data were of high quality for functional annotation and bioinformatic analyses. To validate the RNA-seq data, four genes with different expression levels in the six samples were selectively analyzed using qRT-PCR (Supplemental Table S2). There was a significant correlation between the RNA-seq and qRT-PCR data, with a correlation coefficient of 0.9894 (Figure 2A), confirming the reliability of the transcriptomic data.

After redundancy reassembly, 123,427 unigenes were obtained from all samples, with an average length of 813 nt and an N50 value of 939 nt. After functional annotation, most unigenes (56.3%) were annotated using the NR database, followed by the Swiss (51.9%), GO (50.9%), COG (44.5%), and KEGG (29.3%) databases (Figure 2B). In the COG functional classification, most unigenes were involved in many fundamental functions, including the translation, ribosomal structure and biogenesis, post-translational modification, protein turnover, chaperones, and signal transduction mechanisms (Figure 2C). Based on the functional annotation of KEGG, most of the unigenes were related to the metabolic, signal transduction, and ribosome pathways (Figure 2D). Of all the unigenes, 42.5% were annotated to category A (metabolism), 22.6% were annotated to category E (organismal systems), and 12.7%, 11.2%, and 11.0% were annotated to categories C (environmental information processing), B (genetic information processing), and D (cellular processes), respectively (Figure 2E). GO functional annotation revealed that most unigenes were associated with metabolic processes, cellular processes, intracellular activity, catalytic activity, and binding terms (Figure 2F).

### 2.3. Differentially Expressed Genes and GO Enrichment

**Figure 3:**
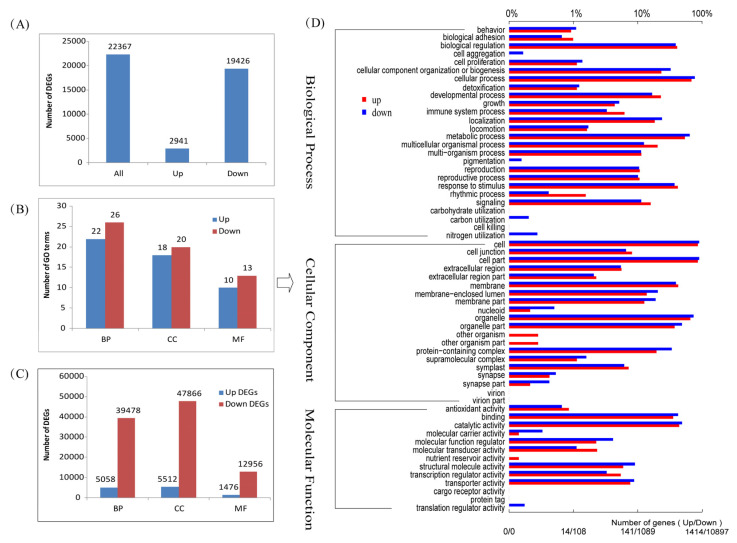
DEGs and GO enrichment analysis in spike-neck of SJ18 compared with those of ZZY. (**A**) Total number of DEGs; (**B**) number of GO terms enriched by DEGs in BP, CC, and FM categories; (**C**) number of DEGs enriched in BP, CC, and FM categories; (**D**) GO enrichment of DEGs and number of genes enriched in each GO term.

Using a two-fold change in gene expression as a screening criterion and ZZY as a control, SJ18 identified 22,367 differentially expressed genes (DEGs), including 2941 upregulated and 19,426 downregulated genes (Figure 3A). GO classification showed that the upregulated genes were enriched in 50 GO terms, including 22 biological process (BP), 10 molecular function (MF), and 18 cellular component (CC) terms, while the downregulated genes were enriched in 59 GO terms, including 26 BP, 13 MF, and 20 CC terms (Figure 3B), which resulted in a total of 39,478 (BP), 47,866 (CC), 12,956 (MF) downregulated DEGs and 5058 (BP), 5512 (CC), 1476 (MF) upregulated EDGs, including some repeated EDGs in different Go terms (Figure 3C). In particular, more GO terms are enriched by downregulated DEGs than those of upregulated DEGs, together with more downregulated DEGs than upregulated DEGs for all enriched GO terms, implying that all GO terms enriched by DEGs showed a downregulated expression trend in SJ18 compared with ZZY (Figure 3D; Supplemental Table S3). These results suggest that there were indeed differences in gene expression patterns between the two varieties and that the low-translocation variety SJ18 possessed a large number of downregulated DEGs compared with the high-translocation variety ZZY; that is, the high-translocation variety ZZY upregulated a large number of DEGs related to plant biological processes, molecular functions, and cellular components. These results may indicate that ZZY is more physiologically and metabolically active than SJ18.

Considering the number of DEGs enriched in each GO term (Figure 3D; Supplemental Table S3), within the BP category, the cellular processes term enriched the largest number of DEGs, amounting to more than 9000, followed by the metabolic process, which enriched more than 7000 DEGs; the terms of biological regulation and response to stimulus enriched more than 4000 DEGs each; the cellular component organization or biogenesis enriched more than 3000 DEGs; the terms of localization and developmental process enriched more than 2000 DEGs; and the five terms of multicellular organismal processes, signaling, multi-organism processes, reproduction, and reproductive processes enriched more than 1000 DEGs. This suggests that SJ18 is not as physiologically active as ZZY in biological processes. Even compared with ZZY, SJ18 was in a lazy state, especially in life processes closely related to Cd stress and transport, such as cellular, metabolism, stress response, and signaling processes, which may be one of the reasons for its low Cd transport efficiency. Analyzing the differences in terms of the CC category between the two varieties, the terms of cell and cell parts were enriched in over 10,000 DEGs; next, the organelle term, organelle part, membrane, protein-containing complex, and membrane-enclosed lumen and membrane part terms enriched more than 8000, 5000, 4000, 3000, and 2000 DEGs, respectively. In the MF category, the catalytic activity and binding terms were enriched in over 5000 DEGs each, and the transporter activity and structural molecule activity terms were enriched in more than 1000 DEGs each. All the above terms were associated with a greater number of downregulated than upregulated DEGs. These results indicate that SJ18 is not as active as ZZY in life processes in terms of cellular components, such as cells, organelles, and membranes, as well as molecular functions, such as catalytic reactions, chelation, and transport dynamics, which may also contribute to the low Cd translocation of the spike-neck in SJ18.

### 2.4. KEGG Enrichment of Differentially Expressed Genes

**Figure 4:**
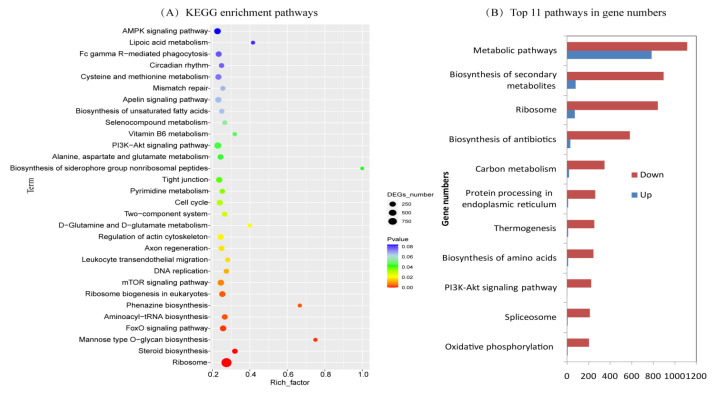
KEGG enrichment of DEGs in spike-neck of SJ18 compared with those of ZZY. (**A**) Top 30 pathways according to their order of significant levels; (**B**) top 11 pathways according to their order of enriched DEGs.

Compared with ZZY, KEGG enrichment identified 21 significantly downregulated pathways in SJ18, all of which showed a much higher number of downregulated DEGs than upregulated DEGs (Figure 4A; Supplemental Table S4). Among them, the downregulated pathways of ribosomes (847 downregulated and 75 upregulated DEGs) suggested that protein synthesis-related processes in SJ18 may be affected by Cd to a greater extent, while exhibiting lower expression levels of most related genes, than ZZY. Meanwhile, for the three downregulated pathways of FoxO, mTOR, and PI3K-Akt signaling in SJ18, the numbers of downregulated genes were 145, 164, and 225, respectively, while the numbers of upregulated genes were below 10, which indicated obvious differences in signaling pathways between the two varieties. Similarly, the steroid biosynthesis, actin cytoskeleton regulation, and cell cycle pathways related to the synthesis of cell membrane and wall substances and senescence were found to be significantly downregulated in SJ18. The pathways of DNA replication and pyrimidine metabolism related to genetic information transfer, together with the pathways of aminoacyl biosynthesis, glutamine, alanine, aspartate, and glutamate metabolism related to amino acid metabolism, were also found to be significantly downregulated in SJ18. These results suggest that SJ18 and ZZY differ in many metabolic pathways and generally show that SJ18 downregulates the expression of numerous DEGs compared with ZZY, which may represent a lower vitality of SJ18 than ZZY.

The same trend was observed in the top 11 KEGG pathways enriched with more than 200 DEGs; that is, the number of downregulated DEGs was much higher than that of upregulated DEGs. These pathways included metabolic pathways, with up to 1906 enriched DEGs (1118 downregulated and 788 upregulated), secondary metabolites biosynthesis pathways involved in multiple life processes, carbon metabolism and oxidative phosphorylation pathways related to energy metabolism, protein processing in endoplasmic reticulum pathways related to protein synthesis, and biosynthesis of amino acid pathways related to amino acid synthesis (Figure 4B; Supplemental Table S4).

### 2.5. WGCNA of DEGs

**Figure 5:**
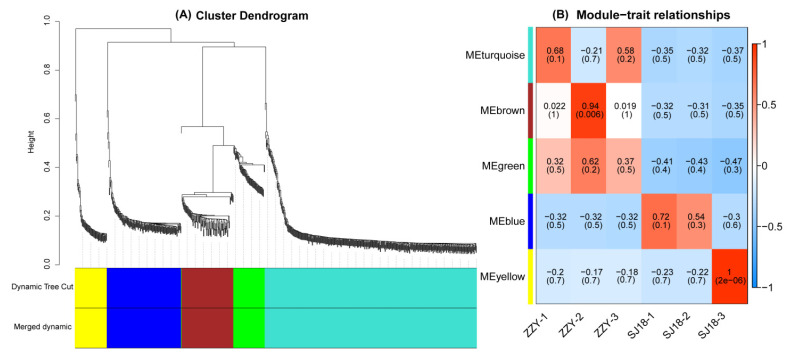
WGCNA of DEGs in spike-neck of SJ18 compared with ZZY. (**A**) Clustering dendrograms of DEGs, with dissimilarity based on topological overlap and the specified module colors; (**B**) module–trait associations between each module and cultivar trait, with each row corresponding to a module eigen gene and each column to a trait, ranging from blue to white to red, indicating low to high correlations.

To identify the main differences in gene expression patterns between rice varieties, we attempted to elucidate the mechanism of the synergistic action of several key pathways in different rice varieties under Cd stress by analyzing the complex co-expression network. Among all DEGs, those with expression levels lower than 1 were removed, and the remaining 799 DEGs were clustered into 5 modules using WGCNA analysis, as shown in Figure 5. Using a kME value above 0.7 as a critical value, 402, 141, 99, 61, and 59 DEGs were collected in the turquoise, blue, brown, yellow, and green modules, respectively (Figure 5A). Although enriched in the largest number of DEGs, the turquoise module did not show a significant correlation with either variety (Figure 5B). Of these modules, the yellow and blue modules showed a correlation with Cd translocation in SJ18, but only the yellow module achieved a significant level (*p* < 0.05); the turquoise, brown, and green modules were associated with Cd translocation in ZZY, while only brown reached a significant level (*p* < 0.05). 

**Figure 6:**
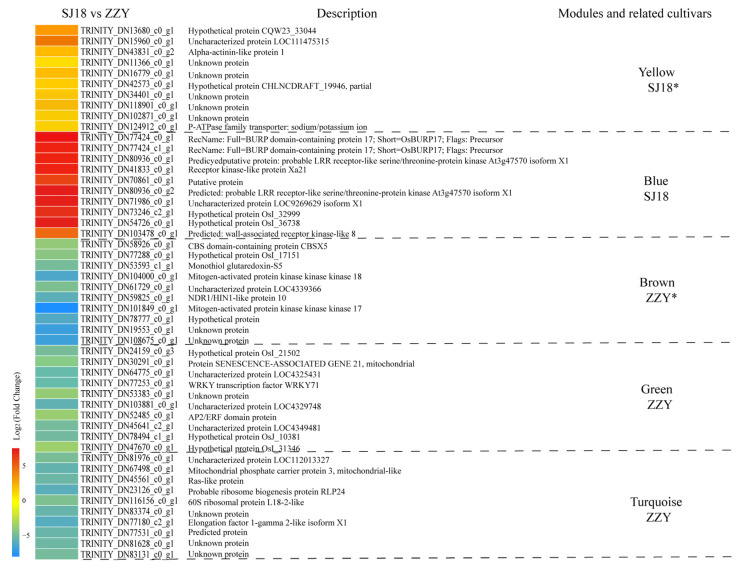
Heatmap of key hub genes in modules of WGCNA analysis of DEGs in the spike-neck of SJ18 compared to those of ZZY. “*” indicates a significant correlation between modules and cultivars traits.

The ten genes with the highest kME values were screened as hub genes for these modules, and the specific kME limit values were 0.9963, 0.9998, 0.9993, 0.9979, and 0.9995 for the yellow, blue, brown, green, and turquoise modules, respectively. The changes in the expression levels and functional descriptions of these genes are shown in Figure 6 and Supplemental Table S5. Overall, compared with ZZY, the hub genes of the yellow and blue modules related to Cd stress in SJ18 showed upregulated expression in the spike-neck of SJ18, whereas the hub genes of the brown, green, and turquoise modules related to Cd stress in ZZY showed downregulated expression in the spike-neck of SJ18; that is, it was upregulated in the spike-neck of ZZY (Figure 6).

Of the 10 hub genes of the yellow module, which were significantly associated with Cd transport in the spike-necks of SJ18, 5 encoded unknown proteins, 3 encoded CQW23_33044, LOC111475315, and CHLNCDRAFT_19946 proteins, which are uncharacterized or hypothetical proteins, and their functions, were not identified; one encoded an alpha-actinin-like protein, and one encoded a P-ATPase family transporter (HMA) protein (Figure 6). For the blue module, which was associated with Cd transport in the spike-neck of SJ18 but did not reach significance, two hub genes encoded BURP proteins, four hub genes encoded receptor kinase-like proteins, and four hub genes encoded proteins with unknown functions, including a putative protein, hypothetical proteins OsI_32999 and OsI_36738, and uncharacterized protein LOC9269629 (Figure 6). From the hub genes of these two modules, it can be hypothesized that the low Cd translocation in the spike-neck of SJ18 may be mainly related to the high expression of HMA and may also include the regulation of cytoskeletal material by the actin network, followed by kinases related to energy metabolism and BURP genes related to abiotic stresses.

The ten hub genes of the brown module, which were significantly associated with Cd transport in the spike-neck of ZZY, included two unknown proteins, two hypothetical proteins, an uncharacterized protein, a cystathionine beta-synthase (CBS) domain-containing protein, a monothiol glutaredoxin, two mitogen-activated protein kinases (MAPKs), and an NDR1/HIN1-like protein. Of the green and turquoise modules that were associated with Cd transport in the spike-neck of ZZY but did not reach significance, 20 hub genes of these 2 modules included 4 unknown proteins, 3 hypothetical proteins, 4 uncharacterized proteins, a predicted protein, a senescence-associated protein, a WRKY transcription factor, an apetala2/ethylene responsive factor (AP2/ERF) domain protein, an RAS-like protein, an elongation factor, a mitochondrial phosphate carrier protein, a probable ribosome biogenesis protein, and a 60S ribosomal protein (Figure 6). Overall, the high Cd translocation in the spike-neck of ZZY may be mainly related to the MAPK signaling process, redox balance, and antioxidant capacities related to CBS and glutaredoxin, and may include the gene NDR1/HIN1.

## 3. Discussion

### 3.1. Down-Regulation of Numerous Physiological Activities May Characterize Low Cd-Transporting Culvitars

Different mechanisms of Cd uptake and transport among different plant species and varieties of the same crop have been a hot topic in the study of Cd tolerance mechanisms in plants with the aim of mining high-quality plant genetic resources. Limiting Cd translocation from stems to grains through node regulation is one strategy for developing low-Cd-accumulating rice varieties [4,18]. Low-Cd-accumulating rice varieties have the ability to limit Cd transport from stems to grains, and the grain Cd content is significantly correlated with the proportion of Cd partitioned between the stems and grains [9,18], which is similar to the findings of this study. We also found that SJ18, a variety with a high spike-neck Cd concentration and low spike-neck–grain Cd transport efficiency, showed a downregulation pattern in many physiological processes compared with ZZY, a variety with a low spike-neck Cd concentration and high spike-neck–grain Cd transport efficiency. These physiological processes are involved in almost all aspects of the plant stress response, such as metabolism, biosynthesis of secondary metabolites pathways, catalytic reactions, chelation, transport dynamics, signaling pathways, energy metabolism, synthesis of cell membrane and wall substances, protein and amino acid synthesis, and genetic information transfer (Figure 3 and Figure 4; Supplemental Tables S3 and S4). Whether these downregulated physiological processes are related to low Cd translocation in SJ18 requires further investigation.

In previous studies, many stress-related pathways, including hormone signaling and transcriptional regulation, were activated in rice under Cd stress, and some pathways related to nutrient reservoirs and starch-related enzymes were inhibited by toxicity [19,20]. Metabolomic studies have revealed that most carbohydrates and amino acids are downregulated by increasing the Cd concentration in rice grains [21]. However, for these inter-variety differences in upward or downward responses to Cd stress, similar to the conclusion of this study, a downregulated ETH signaling pathway and IAA abundance were found in tomato and *Brassica napus* varieties with a high leaf Cd content, respectively [22,23]. Mannitol and cysteine were upregulated, and organic acids, especially those related to alphalinolenic acid metabolism and jasmonic acid production, were activated with an increase in Cd concentration in rice cultivars with low Cd accumulation [21]. However, these upward and downward regulations may be due to different degrees of toxicity symptoms induced by different Cd concentrations or may be related to different Cd transport efficiencies among the varieties. In addition, different genetic backgrounds and resulting metabolic differences among varieties cannot be ruled out, but this study involved a direct comparison of transcriptome data between the two varieties and not a comparison of each variety with a control under Cd stress.

Currently, many studies have focused on the upregulation of metabolic responses related to resistance or tolerance strategies under Cd stress. The upregulation of carbohydrate and amino acid metabolic pathways was observed in the roots of low-Cd-accumulating *Brassica parachinensis* varieties to produce more energy and metabolites for Cd detoxification [24]. The upregulation of cell wall biosynthesis and sulfur and GSH metabolism has also been found in rice cultivars with a strong capacity for Cd retention in the roots [12]. Research has also shown that Cd retained in the roots of low-transport varieties relies mainly on Cd sequestration by cell wall components, especially hemifibers and pectins [10]. The upregulated expression of Cd uptake-related genes has also been found in wheat roots with a high root Cd content and low root–stem Cd translocation efficiency [25]. However, these processes were downregulated in the spike-neck of SJ18, with low transport efficiency and high Cd concentration, which may indicate that the mechanisms of Cd retention in rice roots and spike-necks are different. A previous report also showed that some specific genes exhibited different expression patterns between the roots and leaves in Cd-tolerant tomato cultivars, indicating the presence of tissue-specific responses [26]. However, most current studies have focused on root–stem Cd transport, and less attention has been paid to the stem–spike transport process. Therefore, inter-cultivar differences in rice spike-neck–grain Cd transport processes must be studied in depth.

### 3.2. Different Cd Translocation Efficiencies among Varieties Related to Different Core Mechanisms

We focused on the results of weighted gene co-expression network analysis (WGCNA), especially the modules and hub genes that were significantly related to the tested varieties. The hub genes of the yellow module that were significantly related to SJ18 included a gene encoding HMA, a P-ATPase family transporter and an actin (alpha-actinin-like protein) gene (Figure 6). The effects of OsHMA family genes on Cd enrichment in rice have long been recognized. The overexpression of OsHMA3, a Cd transporter located on the tonoplast, markedly decreased Cd translocation from roots to shoots by enhancing the vacuolar sequestration of Cd in the roots, thereby reducing Cd accumulation in rice grains, and had no significant effect on grain yield or the concentrations of essential micronutrients [27,28]. A highly induced upregulation of the Cd transporter gene HMA3 by Cd stress was found in the roots of *Brassica parachinensis* varieties with low translocation efficiency from roots to leaves [24], which was analogous to the findings of this study, that is, the upregulated expression of OsHMA coincided with the high Cd content and low transport efficiency in the spike-neck of SJ18 and may indicate a more active Cd transport capacity from the apoplast to the vacuole. However, upregulated expression of HMA5 was found in tomato leaves with a low Cd content [23], and high expression of TaHMA2 was found in the roots of wheat varieties with low root Cd and high root–stem Cd transport efficiency, which may indicate that HMA also promotes Cd xylem loading in wheat [25]. Alpha-actinin is a ubiquitous cytoskeletal protein, and the actin network is recognized as a major cytoskeletal system for plant subcellular membrane dynamics [29]. However, the direct influence of Cd stress on actin proteins has not yet been reported. The current results suggest the possible involvement of actin genes in the low-spike-neck–grain Cd transport process of SJ18 by altering the cytoskeletal material composition and its stability. These findings should be followed up with further in-depth studies.

Two MAPK genes appeared as hub genes of the brown module and were significantly associated with ZZY. Most important life processes in plant cells are related to the MAPK-mediated phosphorylation of proteins, including energy metabolism and signaling. Under abiotic stress, protein kinases regulate protein activity and intervene in related cellular responses by catalyzing protein phosphorylation reactions [30]. More than 600 kinase-related genes have been identified in *Arabidopsis*, which play important roles in regulating signal transduction and a variety of metabolic processes; rice is the plant with the largest number of protein kinase genes, which is about twice as many as that of *Arabidopsis* [31]. Previous studies have shown that the MAPK pathway is involved in both biotic and abiotic stresses, including heavy metal stress, by triggering a myriad of transcriptomic, cellular, and physiological pathways in plants [32,33]. The activation effect induced by Cd and Cu stress on the MAPK signaling pathway was found in both *Arabidopsis* seedlings and rice roots [34,35]. Furthermore, the overexpression of OsMAPK5 in transgenic plants can lead to enhanced tolerance to multiple stresses, including drought, salt damage, and cold damage [36]. In addition, a monothiol-glutaredoxin-related gene and a cystathionine beta-synthase (CBS)-related gene were identified as hub genes in the brown module (Figure 6). Monothiol–glutaredoxins play an important role in the redox regulation in plant cells by modulating reactive oxygen species levels through direct scavenging or redox-regulated target proteins to improve a wide range of plant abiotic stresses [37]. CBS also plays key roles in maintaining cellular redox homeostasis by regulating the thioredoxin system during abiotic and biotic stress responses in plants [38], and tobacco plants overexpressing the rice CBSX4 protein gene exhibit salt, oxidation, and heavy metal tolerance [39]. For MAPK, CBS, and glutaredoxin, whose involvement in the plant stress response has been demonstrated, their different expression patterns among cultivars are rarely reported, and whether or not this downregulation in SJ18 spike-necks is related to its low Cd translocation efficiency is unknown, while it is certain that they are significantly correlated with a low Cd content and high Cd translocation in the spike-necks of ZZY. Therefore, it may be possible to investigate approaches to reduce Cd translocation in rice plants from this aspect. In this study, the high Cd concentration, low Cd transport efficiency, and downregulation of glutaredoxin and CBS, two genes related to antioxidant capacity, as well as the MAPK pathway, may have indicated that the antioxidant capacity and signal transduction in the spike-necks of SJ18 were not as strong as those of ZZY, and it may be a manifestation of the toxic effect of high Cd concentrations. Another gene encoding an NDR1/HIN1-like protein was identified as the hub gene of the brown module, which is thought to be involved in plant–pathogen interactions [40], and it is not clear whether the upregulated expression of this gene in ZZY in this study was related to Cd translocation. It is worth stating that although the yellow and brown modules were significantly correlated with the Cd translocation from spike-neck to grains in SJ18 and ZZY, respectively, it has been confirmed that the proteins encoded by the hub genes of these two modules, such as actin proteins, CBS, and NDR1/HIN1-like protein, were related to plant abiotic stress [29,37,38,39]. However, the direct relationship between these hub genes and Cd stress are rarely reported, and these need to be further investigated by proteomics and metabolomics techniques.

In addition to the significantly related modules, the roles of the minor genes cannot be ignored. Among the modules that did not show a significant correlation with any of the test varieties, four kinase-related genes and two BURP genes were identified as hub genes of the blue module (Figure 6), suggesting that these genes play an auxiliary role in the performance of SJ18. For the green and turquoise modules, the hub genes encoding AP2/ERF and WRKY transcription factors, which are involved in protein senescence, oxidative phosphorylation, and ribosomal protein synthesis, also has potential roles affecting the Cd content of ZZY (Figure 6). Studies have confirmed that both WRKY transcription factors and BURP genes play important roles in plant responses to abiotic stress and normal development [41,42] and that StAP2/ERFs are indispensable for Cd uptake and tolerance in potato [43]. Whether these genes are involved in different Cd transport efficiencies between cultivars needs to be confirmed by further studies. Interestingly, 13 of the 20 hub genes in the two modules that were significantly correlated with SJ18 or ZZY, together with 16 of the 30 hub genes in the three modules that did not reach a significant correlation with any test cultivars, encoded uncharacterized, unknown, or hypothetical proteins. This suggests that differences in Cd accumulation or translocation among varieties is a complex process involving multiple factors and requires in-depth mechanistic studies. However, this study only analyzed the differences in RNA-seq data between the two cultivars, and the conclusions of the relationship between these hub genes and cadmium transport efficiency are indeed somewhat abrupt due to lack of functional validation, which will be confirmed by corroboration of gene expression, proteomics, and metabolomics in future studies to enrich our understanding of the molecular mechanisms of cadmium transport in rice. Furthermore, plant root secretions, rhizosphere microbial communities, field water and fertilizer management, soil properties including pH, organic matter, soil texture, and climatic conditions all have a significant impact on the absorption and transport of Cd in rice [5]. The mechanism behind the different Cd uptake and transport between rice varieties caused by the interaction between environmental factors and the genetic background needs to be further investigated.

## 4. Materials and Methods

### 4.1. Experiment Site and Soil Properties 

Field trials were conducted in Masan Town, Yuecheng District, Shaoxing City, which is the main rice-producing area in southern China and a traditional industrial area in the suburbs of Zhejiang Province. The basic physical and chemical properties of the soil included a pH of 5.48, organic matter content of 37.5 g/kg, alkaline nitrogen content of 184.0 mg/kg, available phosphorus content of 36.2 mg/kg, and cation exchange capacity of 17.5 cmol(+)/kg. The main contaminant in the soil was Cd, with a concentration of 0.405 mg/kg. With clay, chalk, and sand contents of 32%, 58%, and 10%, respectively, the soil texture was characterized as silty clay loam.

### 4.2. Rice Cultivars and Field Management

The low and high-grain-Cd-accumulating rice varieties SJ18 (*Oryza sativa* L. *Japonica*) and ZZY (*Oryza sativa* L. *indica-hybrid rice*) were used in the field experiment. Seeds of both varieties were sown in a nursery field, and seedlings approximately 50 days old were transplanted to the test field. The plots were 5 × 8 m in size, and three replicates for each variety were randomly arranged. Local traditional irrigation and fertilization practices were used throughout the rice growth period until harvest and sampling. Briefly, 400 kg/ha of chemical compound fertilizer (15–15–15) was used as a basal fertilizer and applied approximately 15 d before transplanting rice in the field. During the growing period, 50, 50, and 40 kg N/ha of urea were applied as a follow-up fertilizer at the tillering, booting, and filling stages, respectively. Water management was based on the principles of flooding, hydrophobicity, and intermittent flooding during the seedling, tillering, booting, and filling stages. All fertilizers used in the experiments were purchased from local stores.

### 4.3. Sampling and Determination of Cd 

During harvest, five rice plants with three replicates were sampled and divided into roots, stems, grains, flag leaves, and spike-necks, and all samples were dried in an oven at 80 °C for over 5 h. The roots, stems, flag leaves, and spike-neck samples were crushed into powders, and the grains were separated into husks, brown rice, and polished rice powder and stored at 25 ± 5 °C for Cd determination. Additionally, the spike-neck samples for two cultivars with three replicates were collected and frozen in a portable liquid nitrogen tank and then stored at −80 °C for transcriptome analysis.

To determine the total Cd, all plant samples were digested in a HNO_3_/HClO_4_ (5:1 ratio) solution using a microwave digestion system (Mars X, CEM Corporation, Los Angeles, CA, USA). Then, the Cd concentrations were determined by inductively coupled plasma-mass spectrometry (iCAP Q™ ICP-MS, Thermo Fisher Co., Waltham, MA, USA). Reference material GBW10045 was used to ensure data accuracy. The results for the standards were within the allowable uncertainty range with a recovery efficiency of 96.2%.

### 4.4. Transcriptome Analysis of Spike-Neck Samples 

The total RNA of six spike-neck samples was isolated using a TRIzol^®^Reagent kit (Invitrogen, Carlsbad, CA, USA). RNA qualification and quantification, including RNA degradation, purity, concentration, and integrity, were then performed as previously described [17]. Sequencing libraries were constructed using a TruSeq^TM^ RNA sample preparation Kitfrom Illumina (San Diego, CA, USA) with 1 μg of total RNA according tomethods described previously [17]. Briefly, mRNA was isolated by polyA selection using oligo(dT) beads and fragmented using a fragmentation buffer. cDNA synthesis, end repair, A-base addition, and ligation of Illumina-indexed adaptors were performed according to the manufacturer’s protocol. A library size of 200–300 bp of the cDNA target fragment was then selected on 2% low-range ultra-agarose and amplified using Phusion DNA polymerase (NEB) for 15 PCR cycles. After quantifying the end libraries using TBS380 PicoGreen (Invitrogen, Carlsbad, CA, USA), sequencing was performed using a NovaSeq 6000 sequencer (Illumina Inc., San Diego, CA, USA) at Biozeron Co., Ltd. (Shanghai, China).

Raw paired-end reads were trimmed and quality-controlled using Trimmomatic (version 0.36, http://www.usadellab.org/cms/uploads/supplementary/Trimmomatic) (accessed on 2 August 2022), followed by Trinity (http://trinityrnaseq.sourceforge.net) (accessed on 2 August 2022) for de novo RNA assembly using clean data. All assembled transcripts were searched in the NCBI non-redundant protein sequences(NR), a manually annotated and reviewed protein sequence (Swiss), Clusters of Orthologous Groups of proteins (COG), Gene Ontology (GO), Kyoto Encyclopedia of Genes and Genomes (KEGG), and String databases using the BLASTX software (version 2.9.0) to search for proteins with the highest sequence similarity to a given transcript, along with functional annotation. GO annotation of the uniquely assembled transcripts was performed using BLAST2GO (http://www.blast2go.com/b2ghome) (accessed on 10 August 2022). Metabolic pathway analyses were performed using the KEGG (http://www.genome.jp/kegg/) (accessed on 10 August 2022).

Gene expression levels were calculated using the reads per kilobase of exon per million mapped reads (RPKM) method, and EdgeR (http://www.bioconductor.org/packages/2.12/bioc/html/edgeR.html) (accessed on 10 August 2022) was used for differential expression analysis. Differentially expressed genes (DEGs) were defined as those meeting both|log2(Fold Change)|≥1 and false discovery rate (FDR) < 0.05. Functional enrichment of DEGs, including GO and KEGG analyses, was performed to identify the DEGs that were significantly enriched in GO terms and metabolic pathways at a Bonferroni-corrected *p*-value of ≤0.05 compared with a transcriptome-wide background. GO functional enrichment and KEGG pathway analyses were performed using Goatools (https://github.com/tanghaibao/Goatools) (accessed on 17 September 2022) and KOBAS (http://kobas.cbi.pku.edu.cn/home.do) (accessed on 17 September 2022), respectively. In addition, WGCNA was conducted to identify the key modules and hub genes involved in the different Cd transport efficiencies of the spike-necks between cultivars using the BMK Cloud platform (http://www.biocloud.net) (accessed on 10 March 2023).

### 4.5. Quantitative Reverse-Transcription PCR Validation

Four genes with different expression levels between SJ18 and ZZY were selected, and quantitative reverse-transcription PCR (qRT-PCR) was employed to further validate the gene expression levels according to previous methods [17]. The total RNA of the spike-neck was prepared with three replicates by a TRIzol^®^Reagent kit. The transcription of total RNA to cDNA was performed using a PrimeScript™ RT reagent kit with gDNA eraser (Perfect Real Time) purchased from TaKaRa Biotechnology (Dalian Co., Ltd., Dalian, China). Specific primers for each gene were designed using DNAstar software (version 7.1), as shown in Supplemental Table S2. An SYBR^®^ Premix Ex Taq™ Kit (Takara Bio, Dalian, China) and 7900HT Fast Real-Time PCR System (Thermo Fisher Scientific, Waltham, MA, USA) were used to perform quantitative validation. Relative expression was calculated by the 2−ΔΔCT method using GAPDH as an internal control.

### 4.6. Statistical Analysis

Statistical comparisons of the Cd content in various parts of the plant and *t*-tests were performed using Microsoft^®^ Excel 2010 and SPSS version 20.0. Functional annotation maps of the assembled genes, including bar charts, Venn diagrams, scatter graphs, and heatmaps, were constructed using the Cloud Platform software (http://www.cloud.biomicroclass.com/CloudPlatform/home) (accessed on 10 March 2023). WGCNA and its related module–trait associations and clustering dendrograms were produced using the BMK cloud platform (http://www.biocloud.net) (accessed on 22 March 2023).

## 5. Conclusions

Two rice cultivars, ZZY (high spike-neck–grain Cd transport efficiency, low spike-neck and high grain Cd) and SJ18 (low spike-neck–grain Cd transport efficiency, high spike-neck and low grain Cd), were obtained in the field experiment. Comparative analysis of RNA-seq data revealed that, compared with ZZY, 22,367 DEGs were identified in the spike-neck of SJ18. GO analysis enriched 59 downregulated terms, and KEGG enrichment identified 21 significantly downregulated pathways for these DEGs. WGCNA revealed that these DEGs could be clustered into five modules. The hub genes of the yellow module, which were significantly related to SJ18, included OsHMA and OsActin-related genes, whereas those of the brown module, which were significantly related to ZZY, included MAPK, CBS, and glutaredoxin-related genes. This suggests differences in the mechanism of Cd transport from spike-necks to grains among the varieties. The large number of hub genes associated with unknown functional proteins indicates the complexity of Cd transport differences among rice varieties.

## Data Availability

Data are contained within the article.

## Supplementary Materials

The supporting information can be downloaded at: https://www.mdpi.com/article/10.3390/ijms25073592/s1.
